# Cytogenetic analysis of *Phyllomedusa distincta* Lutz, 1950 (2n = 2x = 26), *P*. *tetraploidea* Pombal and Haddad, 1992 (2n = 4x = 52), and their natural triploid hybrids (2n = 3x = 39) (Anura, Hylidae, Phyllomedusinae)

**DOI:** 10.1186/1471-2156-14-75

**Published:** 2013-08-30

**Authors:** Simone Lilian Gruber, Ana Paula Zampieri Silva, Célio Fernando Baptista Haddad, Sanae Kasahara

**Affiliations:** 1 Instituto de Biociências, Departamento de Biologia, UNESP, Universidade Estadual Paulista, Av. 24A, 1515, Rio Claro 13506-900, SP, Brazil; 2 Instituto de Biociências, Departamento de Zoologia, UNESP, Universidade Estadual Paulista, Av. 24A, 1515, Rio Claro 13506-900, SP, Brazil

**Keywords:** Polyploidy, Diploidisation, Chromosome banding, FISH, Molecular cytogenetics

## Abstract

**Background:**

Natural polyploidy has played an important role during the speciation and evolution of vertebrates, including anurans, with more than 55 described cases. The species of the *Phyllomedusa burmeisteri* group are mostly characterized by having 26 chromosomes, but a karyotype with 52 chromosomes was described in *P*. *tetraploidea*. This species was found in sintopy with *P*. *distincta* in two localities of São Paulo State (Brazil), where triploid animals also occur, as consequence of natural hybridisation. We analyse the chromosomes of *P*. *distincta*, *P*. *tetraploidea*, and their triploid hybrids, to enlighten the origin of polyploidy and to obtain some evidence on diploidisation of tetraploid karyotype.

**Results:**

*Phyllomedusa distincta* was 2n = 2x = 26, whereas *P*. *tetraploidea* was 2n = 4x = 52, and the hybrid individuals was 2n = 3x = 39. In meiotic phases, bivalents were observed in the diploid males, whereas both bivalents and tetravalents were observed in the tetraploid males. Univalents, bivalents or trivalents; metaphase II cells carrying variable number of chromosomes; and spermatids were detected in the testis preparations of the triploid males, indicating that the triploids were not completely sterile. In natural and experimental conditions, the triploids cross with the parental species, producing abnormal egg clutches and tadpoles with malformations. The embryos and tadpoles exhibited intraindividual karyotype variability and all of the metaphases contained abnormal constitutions. Multiple NORs, detected by Ag-impregnation and FISH with an rDNA probe, were observed on chromosome 1 in the three karyotypic forms; and, additionally, on chromosome 9 in the diploids, mostly on chromosome 8 in the tetraploids, and on both chromosome 8 and 9 in the triploids. Nevertheless, NOR-bearing chromosome 9 was detected in the tetraploids, and chromosome 9 carried active or inactive NORs in the triploids. C-banding, base-specific fluorochrome stainings with CMA_3_ and DAPI, FISH with a telomeric probe, and BrdU incorporation in DNA showed nearly equivalent patterns in the karyotypes of *P*. *distincta*, *P*. *tetraploidea*, and the triploid hybrids.

**Conclusions:**

All the used cytogenetic techniques have provided strong evidence that the process of diploidisation, an essential step for stabilising the selective advantages produced by polyploidisation, is under way in distinct quartets of the tetraploid karyotype.

## Background

The polyploidy is a process of considerable importance for species evolution and diversification and it has been the aim of multiple reviews [1-3]. Natural polyploidy is widespread in plants, representing one of the most predominant modes of origin of new species or lineages mainly among ferns and flowering plants. Although it is less prevalent in animals, polyploidy occurs in parthenogenetic organisms and in bisexual species or populations. Some examples have been found in insects and among ectothermic vertebrates, such as Salmonidae, Coregonidae, Catostomidae, and Thymallidae fishes, Gekkonidae reptiles, and Caudata and Anura amphibians [1,4]. In anurans, approximately 56 cases of polyploidy have been described in species, populations, and as spontaneously originated polyploid individuals [5-12]. Although the tetraploidy (4x) is relatively common, cases of octoploidy (8x) and even dodecaploidy (12x) have been well documented, such as in the *Xenopus* species [13,14].

In the Hylidae family, the two cases of polyploidy are *Hyla versicolor* (2n = 4x = 48) [15-17] and *Phyllomedusa tetraploidea* (2n = 4x = 52) [18-20]. This latter tetraploid species and the diploid *P*. *distincta* occur in syntopy in some localities of southeast Brazil and there natural interespecific cross produce triploid hybrids [21]. According to Haddad et al. [22], the hybrid individuals were numerous and vigorous, and the advertisement call was indistinguishable from those of the diploid and tetraploid species. Crossing the triploid individuals with the parental species resulted in small egg clutches, low fertilisation rates, and, subsequently, defective tadpoles, and these events occurred in nature because they shared the same breeding site or under experimental conditions [23]. Based on bioacoustics and ecology, Pombal and Haddad [21] suggested autopolyploidy from *P*. *distincta* or *P*. *iheringii* to explain the origin of *P*. *tetraploidea*, but the possibility of allopolyploidy by crossing of *P*. *distincta* with *P*. *iheringii* cannot be discarded. The recent analyses of mitochondrial and nuclear gene sequencing showed that the aforementioned hypotheses are feasible, but in the molecular phylogeny *P*. *distincta* and *P*. *tetraploidea* were sister groups, whereas *P*. *iheringii* appeared in a separate clade [24]. The use of molecular cytogenetic methodologies has been important to contribute for the identification of presumed parental species and, therefore, to enlighten the polyploidy origin in some plants and animal groups, such as in potato [25], fish [26], and salamanders [27].

This work presents data obtained on mitotic or meiotic chromosomes of *P*. *distincta*, *P*. *tetraploidea*, and their triploid hybrids. Additionally mitotic chromosomes of embryos and tadpoles obtained from natural or experimental crosses (3x × 2x and 3x × 4x) are presented. A more detailed characterisation of the karyotypes of the two species and of the hybrids was achieved through classical cytogenetics and more refined approaches, including, for the first time, molecular cytogenetic techniques. The aim was to obtain new information on their chromosome constitution and, eventually, some clues concerning the origin of the polyploidy and the diploidisation in *P*. *tetraploidea* karyotype.

## Results

**Figure 1:**
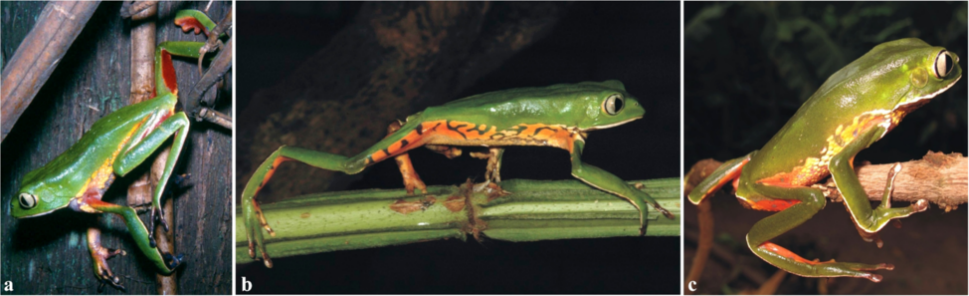
**Adult specimens of *Phyllomedusa***. **a**. *Phyllomedusa distincta* ; **b**. *P*. *tetraploidea*; **c**. triploid hybrid.

**Figure 2:**
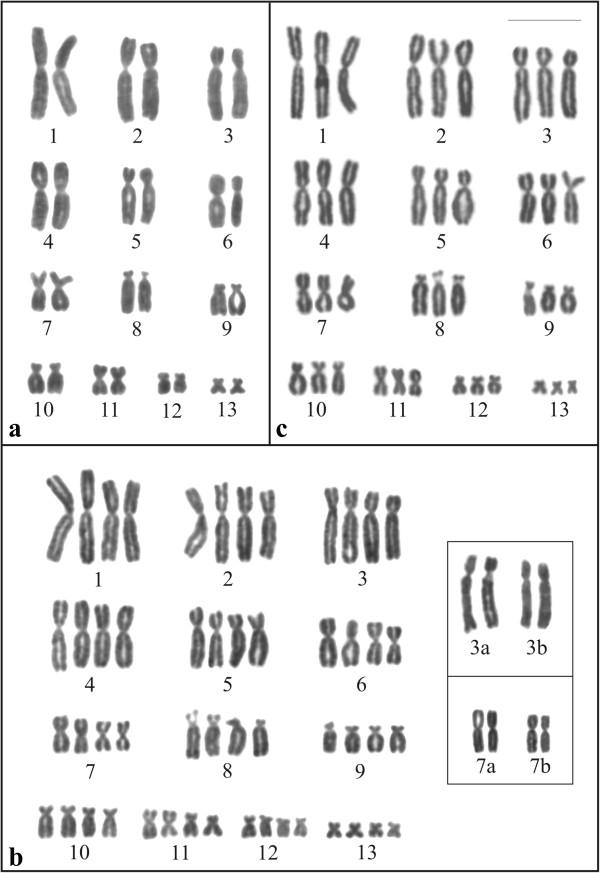
**Giemsa stained karyotypes. a**. *Phyllomedusa distincta*, female, 2n = 2x = 26; **b**. *P*. *tetraploidea*, male, 2n = 4x = 52. Inset: chromosome pair 3a and 3b, and pair 7a and 7b from another specimen; **c**. triploid hybrid, male, 2n = 3x = 39. Bar = 10 μm.

Seven specimens of *Phyllomedusa distincta*, 10 specimens of *P*. *tetraploidea*, and 11 hybrid individuals of these two species (Figure 1, Table 1) were karyotyped. *Phyllomedusa distincta* was 2n = 2x = 26 (Figure 2a), whereas the karyotype of *P*. *tetraploidea* was 2n = 4x = 52 (Figure 2b), and the karyotype of the hybrid individuals of these two species was 2n = 3x = 39 (Figure 2c). The chromosomes were arranged in 13 pairs, quartets, or tercets, respectively, and were metacentric (1, 4, 7, 11, and 13), submetacentric (2, 3, 5, 6, 10, and 12), and subtelocentric (8 and 9). It was observed that in some of the tetraploid specimens, the quartet 3, the quartet 7, or both could be subdivided into two pairs of homologues: chromosome 3a had short arms that were longer than those of chromosome 3b; and chromosome 7a had short arms that were slightly longer than those of chromosome 7b (inset of Figure 2b). Secondary constrictions were occasionally observed at the proximal short arms (p) of chromosome 1 in the three karyotypic forms and in addition at the proximal long arms (q) of chromosome 9 in *P*. *distincta*, at the proximal short arms of chromosome 8 in *P*. *tetraploidea*, and at 8p and 9q in the triploid hybrids.

**Table 1:** List of animals, number and sex of specimens, voucher number, and collection site in Brazil

**Animals**	**Number of specimens**	**Sex**	**CFBH**	**Collection site**
*Phyllomedusa distincta* (2x)	1	male	13558	Guaraqueçaba, PR
	4	male	2460, 2471, 2577, 6912	Ribeirão Branco, SP
	1	female	1810	
	1	male	33315	Ribeirão Grande, SP
*Phyllomedusa tetraploidea* (4x)	7	male	2110, 2112, 2639, 2718, 2721, 6920, 6925	Ribeirão Branco, SP
	1	female	2464	
	1	male	33314	Ribeirão Grande, SP
	1	female	2599	Tatuí, SP
Triploid hybrid (3x)	10	male	1730, 2050, 2111, 2463, 2470, 2638, 2719, 2722, 2723, 2728	Ribeirão Branco, SP
	1	male	33316	Ribeirão Grande, SP

PR: state of Paraná; SP: state of São Paulo.

CFBH: collection Célio F.B. Haddad, housed in Instituto de Biociências, UNESP, Rio Claro, SP, Brazil.

Guaraqueçaba: 25°17′25“S; 48°18′55”W; Ribeirão Branco: 25°17′25“S; 48°18′55”W; Ribeirão Grande: 24°551“S; 48°22′19”W; Tatuí: 23°20′25“S; 47°51′23”W.

**Figure 3:**
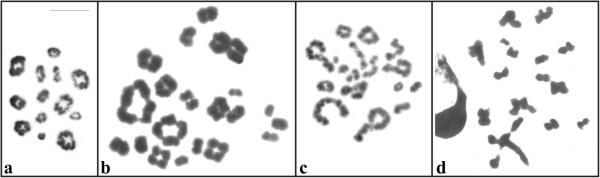
**Giemsa stained meiotic cells from males. a**. *Phyllomedusa distincta*; **b**. *P*. *tetraploidea*; **c**, **d**. triploid hybrid. **a**, **b**, **c**. cells in initial phases; **d**. cell in metaphase II. Bar = 10 μm.

The meiotic analyses carried out in the male specimens of *P*. *distincta* identified 13 bivalents during the diplotene, diakinesis, and metaphase I cells (Figure 3a) and 13 chromosomes in metaphase II (data not shown). The male specimens of *P*. *tetraploidea* possessed a variable number of tetravalents and bivalents in the diplotene, diakinesis, and metaphase I cells (Figure 3b) and 26 chromosomes in metaphase II (data not shown). In the testis preparations of triploid males, early meiotic phases were observed, likely in diplotene, diakinesis, and metaphase I cells, with univalent, bivalent or trivalent chromosomal configurations (Figure 3c); cells in metaphase II (Figure 3d) possessed a variable number of chromosomes; and spermatids in process of differentiation were visualised.

**Figure 4:**
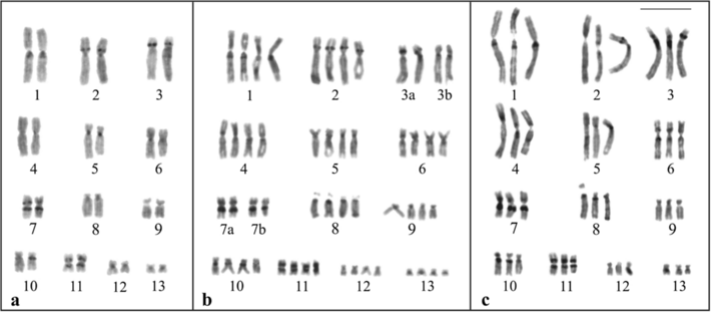
**C-banded karyotypes. a**. *Phyllomedusa distincta*; **b**. *P*. *tetraploidea*; **c**. triploid hybrid. In b, the quartets 3 and 7 are subdivided into pairs of homologous chromosomes. Bar = 10 μm.

C-banding was performed on cytological preparations of three specimens of *P*. *distincta*, four of *P*. *tetraploidea*, and three triploid hybrids. The distribution of C-banded heterochromatin in the pair, quartet, and tercet of chromosomes was approximately equivalent (Figure 4a-c). Chromosomes 2, 3, 4, 5, 8, 9, 12, and 13 possessed centromeric bands, but some chromosomes exhibited a tiny or almost absent C-band. The remaining chromosomes, 1, 6, 7, 10, and 11, had large pericentromeric blocks, and depending on the chromatin condensation, these blocks continuously extended to the centromeric band, or they appeared as clearly separated bands in the proximal region of the short arms, the proximal region of the long arms, or both. Chromosomes 2 and 3 in the three karyotypic forms exhibited additional interstitial C-bands in the short arms that were very prominent in some metaphases. Interestingly, the C-bands were not always identical among the homologous chromosomes within each pair, quartet, or tercet. Nevertheless, for some tetraploids, including the specimen depicted in Figure 4b, quartet 3 could be subdivided, presenting a conspicuous interstitial C-band in the short arms of chromosome 3a that was not observed in chromosome 3b, and quartet 7 exhibited slightly different C-banding and could also be subdivided into two pairs, with a pericentromeric block in the short arms of chromosome 7a that was larger than the block observed in chromosome 7b.

**Figure 5:**
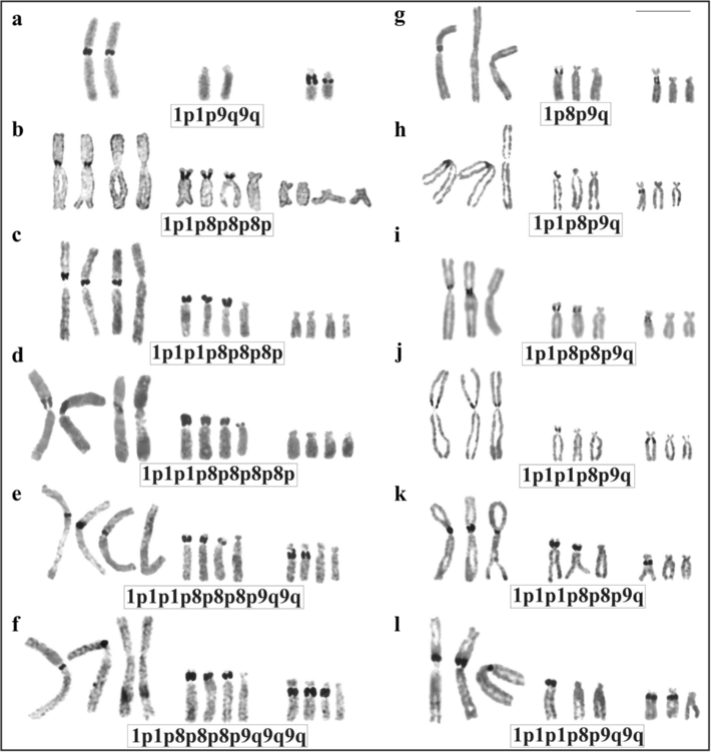
**Chromosomes 1, 8, and 9 with distinct patterns of Ag-NORs. a**. *Phyllomedusa distincta*; **b**, **c**, **d**, **e**, **f**. *P*. *tetraploidea*; **g**, **h**, **i**, **j**, **k**, **l**. triploid hybrid. Bar = 10 μm.

The chromosome preparations of the sampled *P*. *distincta*, *P*. *tetraploidea*, and triploid hybrids, with exception of two tetraploids and one triploid specimen, were analysed with silver impregnation. The Ag-NOR in *P*. *distincta* was on both homologues of pair 1 (1p) and on both homologues of pair 9 (9q), according to the 1p1p9q9q pattern (Figure 5a). In *P*. *tetraploidea* the Ag-NOR was present on chromosome 1 (1p) and on chromosome 8 (8p). In six of the specimens of the sample, the total of silver impregnated sites ranged from four to eight per metaphase (Figure 5b-d) but this number was relatively invariable in the sampled cells for each individual, with exception of one specimen that showed intraindividual variation of Ag-NORs. In two other specimens, Ag-NORs were also observed on chromosome 9 (9q), according to the 1p1p1p8p9q9q pattern (figure not shown) in one case or the 1p1p1p8p8p8p9q9q and the 1p1p8p8p8p9q9q9q patterns in the other (Figure 5e-f). In the triploid hybrids Ag-NOR was present at 1p, 8p, and 9q and the total number of Ag-NORs ranged from three to six per metaphase but was relatively invariable within the sampled cells of each individual (Figure 5g-k). One hybrid triploid exhibited the 1p1p1p8p9q9q Ag-NOR pattern, with two silver impregnated chromosomes 9 (Figure 5l).

**Figure 6:**
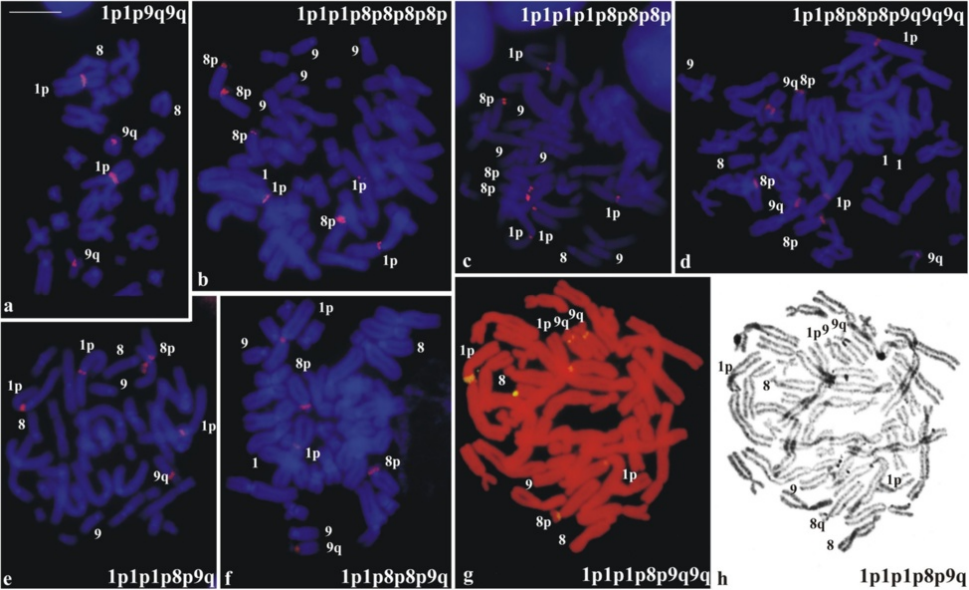
**Metaphases with distinct patterns of NORs.** FISH using an rDNA probe **(a-g)** and Ag-impregnation **(h)**, the same shown in **(g)**. **a**. *Phyllomedusa distincta*; **b**, **c**, **d**. *P*. *tetraploidea*; **e**, **f**, **g**, **h**. triploid hybrid. Bar = 10 μm.

The FISH technique with an rDNA probe was performed on cytological preparations from three individuals of each karyotypic form. In the representatives of *P*. *distincta*, four hybridisation signals per metaphase were always observed and followed the 1p1p9q9q pattern (Figure 6a). The specimen of *P*. *tetraploidea* that showed Ag-NOR intraindividual variation exhibited 1p1p8p8p8p8p-type hybridisation pattern in all of the metaphases (Figure 6b); the specimen that possessed the 1p1p1p8p8p8p Ag-NOR pattern exhibited 1p1p1p1p8p8p8p-type hybridisation pattern (Figure 6c); and one specimen that possessed 1p1p1p8p8p8p9q9q and 1p1p8p8p8p9q9q9q Ag-NOR patterns exhibited 1p1p8p8p8p9q9q9q-type hybridisation pattern (Figure 6d). The triploid hybrid that possessed the 1p8p9q Ag-NOR pattern exhibited 1p1p1p8p9q-type hybridisation pattern (Figure 6e); the specimen that possessed 1p1p8p8p9q Ag-NOR pattern exhibited 1p1p8p8p9q-type hybridisation pattern (Figure 6f); and one specimen that possessed 1p1p1p8p9q Ag-NOR pattern (Figure 5j) exhibited 1p1p1p8p9q9q-type hybridisation pattern (Figure 6g). In this latter specimen, the silver impregnation technique was also performed sequentially after FISH with an rDNA probe (Figure 6h), and the metaphases followed the 1p1p1p8p9q Ag-NOR pattern, as it had been previously observed in the cytological material not treated before with any procedure other than the silver impregnation.

**Figure 7:**
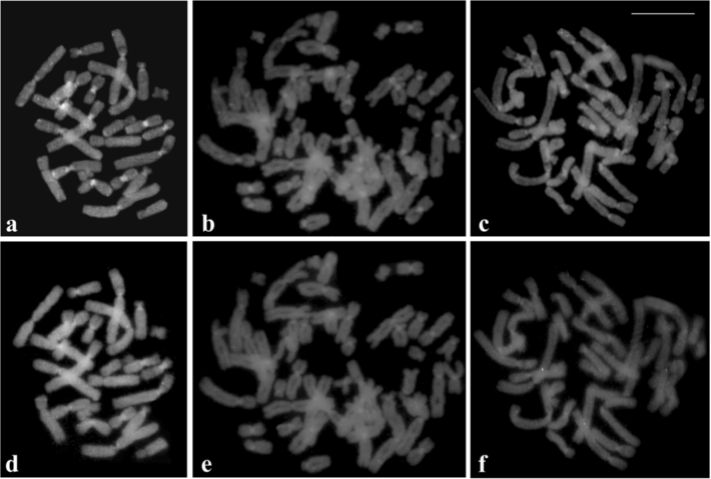
**Fluorochrome stained metaphases.** DA/CMA_3_**(a, b, c)** and DA/DAPI **(d, e, f)**. **a**, **d**. *Phyllomedusa distincta*; **b**, **e**. *P*. *tetraploidea*; **c**, **f**. triploid hybrid. Bar = 10 μm.

The cytological preparations of two specimens of *P*. *distincta*, two specimens of *P*. *tetraploidea*, and four triploid hybrids were stained with base-specific fluorochromes. CMA_3_ used with DA counterstaining produced bright centromeric fluorescence in almost all of the chromosomes, with the exception of chromosome 13 of the smallest group in the complement of the three karyotypic forms (Figure 7a-c). After DAPI with DA counterstaining, almost all chromosomes were slightly fluorescent in the centromeric region (Figure 7d-f).

**Figure 8:**
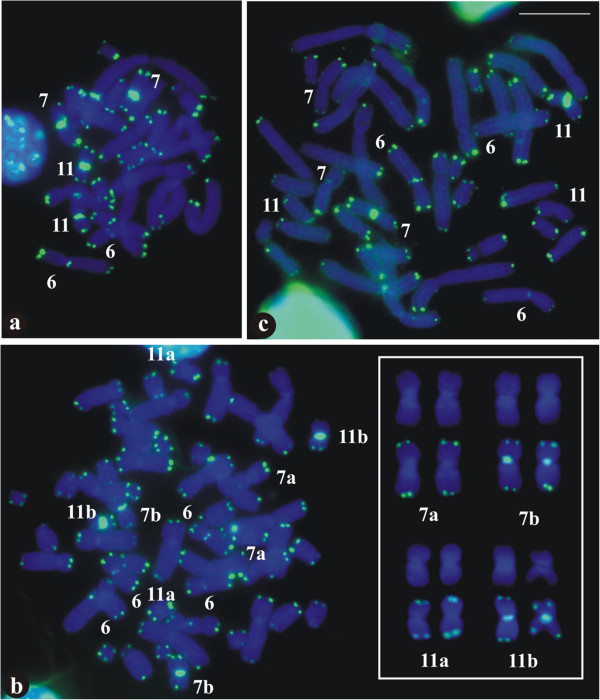
**Metaphases with FISH using a telomeric probe. a**. *Phyllomedusa distincta*; **b**. *P*. *tetraploidea*; **c**. triploid hybrid. Note additional hybridisation signal in the centromeric region of some chromosomes: two 7 and two 11 in **a** and **b**; one 7 and one 11 in **c**. Inset: chromosome pairs 7a and 7b, and 11a and 11b from another metaphase with DAPI staining and hibridised with the telomeric probe. Bar = 10 μm.

The FISH technique using the telomeric probe identified terminal regions of all of the chromosomes in the three karyotypic forms (Figure 8a-c). In the metaphases of *P*. *distincta*, the chromosome pairs 7 and 11 exhibited additionally strong hybridisation signals in the centromeric region (Figure 8a), whereas in of *P*. *tetraploidea*, two chromosomes 7 and two chromosomes 11 exhibited interstitial centromeric signals (Figure 8b). The chromosomes 7a and 7b as well as the chromosomes 11a and 11b, from another metaphase, with DAPI staining and hybridised with the telomeric probe, are shown in the inset of Figure 8b. In the triploid hybrid the centromere of one chromosome 7 and one chromosome 11 was additionally hybridised with telomeric probe. In the three karyotypes, chromosome 6 also exhibited a small hybridisation signal in the centromeric region (Figure 8c).

**Figure 9:**
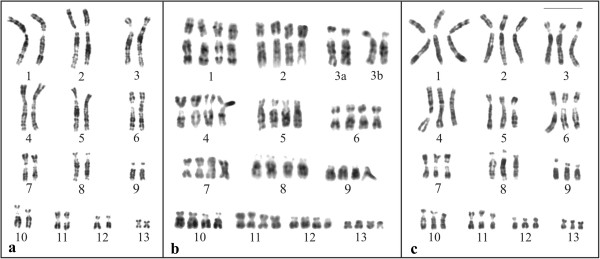
**Replication banding after BrdU incorporation. a**. *Phyllomedusa distincta*; **b**. *P*. *tetraploidea* with the quartet 3 subdivided into pairs 3a and 3b; **c**. triploid hybrid. Bar = 10 μm.

Treatment with BrdU in two specimens of *P*. *distincta*, two specimens of *P*. *tetraploidea*, and four hybrids produced relatively good replication bands, which allowed the correct identification of each group of homologous chromosomes (Figure 9a-c). The chromosomes in the three karyotypic forms showed a high degree of homeology. In the banded karyotype of one tetraploid specimen (Figure 9b), the chromosomes of quartet 3 could also be subdivided into pairs 3a and 3b, according to differences in their short arms.

**Figure 10:**
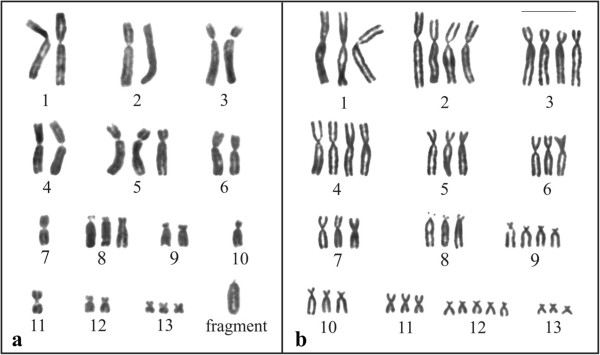
**Giemsa stained karyotypes. a**. embryo obtained in the laboratory, derived from cross between triploid and diploid specimens, with 26 chromosomes + fragment. **b**. tadpole collected in nature, most likely derived from cross between triploid and tetraploid specimens, with 45 chromosomes. Bar = 10 μm.

The analyses of the cytological preparations obtained from some embryos, tadpoles, and one newly metamorphosed animal in the laboratory (A113) showed variable chromosome number, so the modal karyotype could not be established. A balanced chromosome constitution was never observed, and even when an identical chromosome number was present in the sampled cells of the same analysed specimen, the karyotypes were distinct. The karyograms of two individuals, most likely derived from crosses between parental 3x and 2x or 3x and 4x are shown in Figures 10a and b, respectively.

## Discussion

*Phyllomedusa* specimens of this study share the same basic set of chromosomes (x = 13) and the morphology in each pair, quartet, and tercet of chromosomes from *Phyllomedusa distincta*, *P*. *tetraploidea*, and the triploid hybrids, respectively, is equivalent among them and does not deviate from the majority of species analysed thus far, indicating a high karyotypic conservation within the genus. Among 19 species of *Phyllomedusa* cytogenetically analysed, only *P*. *tetraploidea* is polyploid [15,28-30]. All of the remaining species are diploid with 26 bi-armed chromosomes, with the exception of *P*. *tarsius*[31] and *P*. *camba*[32], bearing some uni-armed telocentric pairs, and a male of *P*. *rohdei* from Linhares, in the Espírito Santo State (Brazil) [33] with 2n = 40 karyotype and a probable XY pair, although animal samples from this same locality and from other regions of southeast and northeast Brazil [20,32,34] exhibited a 2n = 26.

With C-banding, equivalence of the basic chromosome set is also true since the karyotypes of *P*. *distincta*, *P*. *tetraploidea*, and the triploid hybrids had approximately similar distributions of heterochromatin. Interestingly, the elements in each pair, quartet, and tercet were characterised by a particularly distinctive C-banding pattern, so the homologues within each of these groups could be precisely identified. Batistic [20] had previously reported similar C-banding patterns in the three karyotypic forms and argued that some differences among the homologues in the chromosome groups resulted from differential chromatin condensation or from a variable response to the banding treatment. Although both conditions are possible, the homologues in both quartets 3 and 7, shown for the tetraploid specimen in Figure 4b, were subdivided into two pairs (3a and 3b, 7a and 7b) that highly suggest diploidisation. In C-banded metaphases from other tetraploid individuals, subdivision was apparent exclusively for quartet 3 or for quartet 7, or none of them. Therefore, we conclude that although two types of chromosome 3 and two types of chromosome 7 exist in the tetraploid population, they do not always segregate as homologous pairs. This explains the slight variable tetraploid karyotype constitutions analysed with standard staining. Further analyses of C-banding in a larger number of *P*. *tetraploidea* would be relevant to support the hypothesis that evolution towards a diploid constitution is underway in the tetraploid *Phyllomedusa* species.

Unlike most hylids carrying one Ag-NOR [15,35-37], *Phyllomedusa* species, with some exceptions, generally exhibited multiple sites and variability in the number and location of the Ag-NOR sites has been observed [20,28-30,32-34,38]. At least three of the five known species of the *P*. *burmeisteri* group, i.e., *P*. *burmeisteri*, *P*. *distincta*, and *P*. *iheringii* with the exception of *P*. *bahiana*, which is not yet karyotyped, have conservatively Ag-NORs on chromosomes 1 and 9. *Phyllomedusa tetraploidea*, which belongs to the same *P*. *burmeisteri* group, only shares Ag-NOR located on chromosome 1, and apparently, the nucleolar organiser moved from chromosome 9 to 8, most likely during the differentiation of the tetraploid karyotype. Nevertheless, our data both with Ag-impregnation and FISH with an rDNA probe showed that chromosome 9 unequivocally carrying rDNA regions was also found in some few tetraploid individuals and some hypotheses can be pointed out: the marker chromosome 9 was not completely lost from the tetraploid population; alternatively, segments with few copies of ribosomal genes remained at the original NOR site on chromosome 9 during the polyploidisation process, and they could have been amplified under certain conditions; and finally, introgression of an ancestral diploid character occurred in a tetraploid population via interspecific hybrids.

In *P*. *distincta* there was no evidence of inactive nucleolar organiser region or occurrence of chromosome 1 or 9 not bearing ribosomal sequence because the specimens consistently exhibited four NORs identified by Ag-impregnation and an rDNA probe. The variability observed with both techniques in the sample of *P*. *tetraploidea* might be explained assuming the genetic inactivity of some sites, the difficulty in visualising some small signals, or overlapping chromosomes. Nevertheless, instead of inactivating the ribosomal genes in chromosome 1 or 8 by gene regulation, the loss of rDNA sequences seems to be frequent and well-tolerated in the karyotype of *P*. *tetraploidea* because the necessary gene product is guaranteed. The existence of chromosomes 1 and 8 lacking ribosomal genes in *P*. *tetraploidea* might preliminarily indicate the reorganisation of the tetraploid genome toward the process of diploidisation. Among the hybrid *Phyllomedusa* specimens, the observed Ag-NOR patterns are in accord with the expected results from the crosses between *P*. *distincta* and *P*. *tetraploidea* and the finding of one triploid specimen bearing two chromosomes 9 with silver impregnated site is not surprising because marker chromosome 9 had already been registered in the tetraploid population. The FISH technique fully confirmed the Ag-NOR pattern and in one of the specimens with more than one chromosome 9 bearing rDNA sequences, which one of them is not transcriptionally active.

The chromosomes of *P*. *distincta*, *P*. *tetraploidea*, and the hybrids showed no obvious differences in the fluorochrome stainings, but some repetitive regions did have variable molecular compositions, although this was not very clear in the majority of the metaphases. Both fluorochromes remarkably produced fluorescence in the centromeric region of the chromosomes, although less brilliant with DAPI, suggesting the existence of repetitive DNA enriched with either GC or AT. In the centromere region of chromosomes 7 and 11 of *P*. *distincta* and *P*. *tetraploidea*, there was also indication of telomere-like sequences, as it has been observed in other hylids [36,37] not representing vestiges of true telomeres after possible structural rearrangements [39,40]. In the *P*. *tetraploidea* specimen, the interstitial centromere signal in chromosomes 7 and 11 might additionally indicate an early diploidisation.

The replication bands confirmed the high degree of homeology regarding the chromosomes of *P*. *distincta*, *P*. *tetraploidea*, and their triploid hybrids. Nevertheless, the banding patterns of the homologues within each pair, tercet, or quartet are not absolutely identical, and we cannot completely exclude the possibility that they resulted from normal asynchronies during the replication process or were technical artefacts. In some cases, such as in the quartet 3 of the tetraploid karyotype shown in Figure 9b, the subdivision into two chromosome pairs reinforces the hypothesis of diploidisation revealed by the C-banding pattern.

The tetravalent formation in *P*. *tetraploidea* males (Figure 3b) could at first sight indicate autopolyploidy, but considering the high degree of chromosomal conservation in the species of the genus, allopolyploidy cannot be excluded as a possibility. The triploid hybrids showed initial meiotic stages where chromosomes had degenerate aspect but a few cells had relatively normal appearance. Surprisingly, some spermatids, perhaps those carrying approximately balanced chromosomal constitutions, are able to undergo subsequent differentiation into spermatozoa, which explain the fertility of the hybrid specimens. According to the observations in the field that were corroborated in the laboratory experiments [22,23], the triploid hybrids mated in nature with diploids, tetraploids, or triploids, but the egg clutches had a low number of eggs or low fertilisation rate. Some of these eggs often produced small, unpigmented, and severely physically deformed tadpoles unable to complete development; however, one karyotyped individual metamorphosed in the laboratory during this study. We conclude that the zygotes with approximately normal chromosome constitutions, among the thousands of zygotes, formed in nature from one or two triploid parents, are able to initiate embryonic development. During this process, aleatory chromosome elimination might occur during cellular divisions, but only the cells possessing nearly balanced chromosome constitutions would survive. The crossings between triploid partners mostly likely produce non-viable offspring.

The diagnosis of the ploidy level in *Phyllomedusa* representatives was based preliminarily on the body size and the colour patterns of the inner thigh [23]. According to this author, the triploid specimens were generally slightly larger than the tetraploids, which, in turn, were larger than the diploids, and although the hybrids exhibited intermediate thigh colour in general, some of them shared the colour pattern of one of their parents. Therefore, determinating the mitotic chromosome constitution, even using only standard staining, and observing the meiotic phases are of fundamental importance to unequivocally establish the ploidy level of the animals.

The origin of a polyploid species is often a matter of interest, and it has been investigated through molecular cytogenetic techniques. In the *Primula* plant, the GISH technique was used to demonstrate the hybrid origin of the polyploid [41], whereas in the unisexual *Ambystoma* salamanders, the parental species of different polyploids and the hypothesis on the events involved in the origin of some forms could be advanced [27]. In the African clawed frogs complex of *Xenopus* and *Silurana* genus, *S*. *tropicalis* (2n = 20) is the only known diploid species [42]. Microdissected chromosomes of this species were used for chromosome painting in the *Xenopus laevis* (2n = 4x = 36) metaphases, confirming its polyploid status and explaining the origin of both *Xenopus* and *Silurana* genera [42], and, later, the chromosome homeologies between *X*. *laevis* and *S*. *tropicalis* were corroborated by gene mapping [43].

The diploidisation is an important step in the evolution of polyploid karyotypes for stabilising the selective advantages produced by polyploidisation and it has been described as a natural evolutionary pathway, whereby a disomic status is reestablished. According to Ohno [44], diploidisation is favoured by the preferential formation of bivalents during meiosis, which would be eased by structural changes in the chromosomes that allow the functional diversification of the homologues. This process usually includes the successive accumulation of structural changes, mainly involving repetitive regions [45]. Diploidisation can be cytologically recognised when the chromosomes within the same group present distinct patterns indicating a change in the quantity, distribution or position of repetitive sequences. For example, the loss of rDNA cistrons, as observed in the octoploid *Ceratophrys ornata* anuran, where two octets included only half of their chromosomes bearing NORs [46], and in plants of *Primula* and *Iris* genera [41,47], was considered evidence of the diploidisation process. Eventually, this phenomenon was identified by C-banding or other cytogenetic techniques, as in the tetraploid *Odontophrynus americanus* anuran [46]. In *P*. *tetraploidea*, it was possible to ascertain diploidisation by our highly accurate analysis that revealed differences in the morphology of standard stained chromosomes, in quantity and distribution of C bands, in ribosomal sequences, and in the presence of interstitial telomere-like sequences. Although diploidisation has been observed on quartets 1, 3, 7, 8, and 11, this process most probably occurs in other quartets.

Polyploidy has great evolutionary importance because of the potential for rapid speciation, without the occurrence of transitional forms. The duplication of the genomic content may provide an adaptive advantage for the new species because gene duplication creates new gene loci, and this is critical to the successful establishment of the new polyploid lineage to adapt to the environment. Furthermore, the polyploidisation may create an increased and more durable heterosis, and it may lead to the loss of sexual compatibility or the gain of asexual reproduction [48]. The genome polyploidisation in the case of *P*. *tetraploidea* actually represented an advantage, allowing the occupation of new environments in the plateau area, where the animals are subjected to greater seasonality compared to the other species of the *P*. *burmeisteri* group that are distributed in the coastal regions [22].

## Conclusions

Considering that polyploidy is a rapid speciation process, the new polyploid species still retains high chromosome homeology with the parental species and this seems to be the case of *P*. *tetraploidea*. Our present data could not clarify the origin of the polyploidy in *P*. *tetraploidea*, although diverse techniques had been used. New cytogenetic approaches are still necessary to enlighten the origin of the tetraploidy in *Phyllomedusa* and, in this case, the analyses should include other species of the genus. The standard and differential cytogenetic techniques undoubtedly revealed new information concerning *Phyllomedusa*, especially regarding the evidence of diploidisation in the tetraploid karyotype. Certainly, the diploidisation is an ongoing process in *P*. *tetraploidea*.

## Methods

Cytogenetic analyses were performed on 28 adult specimens of *Phyllomedusa* from localities in the states of São Paulo and Paraná (Table 1, Figure 1). The animals were identified by one of the researches (CFBH). The examined specimens were deposited in the amphibian collection (CFBH of the Departmento de Zoologia, Instituto de Biociências, UNESP, Rio Claro, SP, Brazil). We also analysed the metaphases of eight tadpoles and of a single specimen (A113) that metamorphosed in the laboratory, from two egg clutches collected in Ribeirão Branco, SP, resulting from unknown crosses possibly occurred between a triploid hybrid and *P*. *tetraploidea*. We analysed the chromosomes from three tadpoles as well as four embryos from two egg clutches obtained by experimental crosses between a triploid hybrid and *P*. *distincta* or a triploid hybrid and *P*. *tetraploidea*. The research was conducted in accordance with Brazilian legislation governing standards of ethical procedures for collecting and scientific studies, and under consent and approval of Instituto Brasileiro do Meio Ambiente e dos Recursos Naturais Renováveis (IBAMA - permission 23129-1).

Direct chromosome preparations of adult specimens were obtained from the bone marrow, liver, and intestinal epithelium as well as the testes from males [49,50]. For some animals, the cell suspensions were additionally obtained from lymphocyte cultures [51]. In vivo [52] or in vitro [51] treatment with 5-bromodeoxyuridine (BrdU) was performed in some animals.

Standard staining of the chromosomes was performed with Giemsa diluted in phosphate buffer pH 6.8, silver impregnation of the nucleolus organiser regions (Ag-NOR) followed the technique described by Howel and Black [53], and the identification of heterochromatic regions (C-bands) followed Sumner [54]. To differentiate the replication bands, the Fluorochrome Plus Giemsa techniques described by Dutrillaux and Couturier [55] or Matsuda and Chapman [56] were used. Simultaneous staining with the AT-specific fluorochrome 4′, 6-diamidino-2-phenylindole (DAPI) and GC-specific chromomycin A_3_ (CMA_3_) with distamycin A (DA) counterstaining was used according to the technique described by Schweizer [57]. Fluorescent in situ hybridisation (FISH) with the ribosomal probe HM123 [58] was performed according to the technique of Pinkel et al. [59], and the hybridisation of the telomeric probe from the DAKO kit was performed following the manufacturer’s manual (Dako Cytomation Denmark A/S Kit).

To obtain chromosomal preparations from embryos, the embryos were kept for approximately 4 hours in Hanks solution diluted 1:1 in water with colchicine added to a final concentration of 0.1%. The embryos were incubated in hypotonic solution prepared with frog saline and distilled water (1:15) for 30 minutes at 37°C and dissociated in methanol and acetic acid fixative (3:1). The tadpoles were placed in an aquarium containing colchicine solution at a final concentration of 0.1% for 4 hours. To obtain the cell suspensions, the tip of the tail and the intestines were removed and incubated in a hypotonic solution of 0.075 M potassium chloride for 30 minutes at 37°C. The tissues were dissociated in a methanol and acetic acid fixative (3:1). From the animal that metamorphosed in the laboratory, only the intestine was used, as described for tadpoles. For all of the samples, we performed several fixative washes before slide preparation. The chromosomes from the embryos and tadpoles were only analysed with standard Giemsa staining.

The chromosomes were analysed under light microscopy or UV light. The cells were photographed with black and white Kodak Image Link or T-Max film and with Kodak ISO 400 colour film. The copies were printed on Kodak photographic paper or on ordinary paper from the scanned negative. Some of the images of the analysed material were captured digitally. The karyograms were constructed according to the morphology of the chromosomes and in descending order by size. The bi-armed chromosomes of the karyotypes were classified by visual inspection, as metacentric, submetacentric, or subtelocentric [60,61].

## Abbreviations

2n: Diploid number; Ag-NOR: Nucleolar organiser region marked by silver impregnation; BrdU: 5-bromodeoxyuridine; CMA3: Chromomycin A_3_; DA: Distamycin A; DAPI: 4′-6-diamidino-2-phenylindole; FISH: Fluorescent in situ hybridisation; FPG: Fluorochrome plus Giemsa; NOR: Nucleolar organiser region; rDNA: Ribosomal DNA.

## Competing interests

The authors declare that they have no financial competing interests.

## Authors’ contributions

SLG performed the cytogenetic studies and drafted the manuscript. APZS performed some cytogenetic studies and revised the manuscript. CFBH collected and identified the animals, provided support on zoological information, and revised the manuscript. SK supervised the cytogenetic studies, participated in the draft, and in the revision of the final text. All authors read and approved the final manuscript.
